# Factors associated with poor mental health during mandatory home work: a cross-sectional study in university staff

**DOI:** 10.1186/s12995-023-00382-6

**Published:** 2023-07-28

**Authors:** Philippe Kiss, Marc De Meester, Ilse Vingerhoets, Bart Garmyn, Annemie Raemdonck

**Affiliations:** 1 Securex Occupational Health Service, Gent, Belgium; 2 Department of Public Health and Primary Care, Ghent University, Ghent, Belgium

**Keywords:** Mental health, Mandatory home work, University staff, Need for recovery, Burnout symptoms, Work-private life balance

## Abstract

**Background:**

During the first wave of the Covid-19 pandemic in 2020, a total lockdown of universities was implemented by the government in Belgium. University staff was required to work at home. The purpose of the study was to identify factors associated with poor mental health in university staff during mandatory home work.

**Methods:**

Mental well-being of 702 university employees was assessed by need for recovery after work and presence of burnout symptoms. Following factors were considered: personal factors (gender, age), professional status, specific home work environment factors (quiet place to work, taking care of ill or old people, number of children < 12yrs. at home, family member at risk for Covid-19), work-private life balance and worries about long- and short-term work situation. Multivariable logistic regression analyses were used to calculate the odds ratios for the presence a high need for recovery and burnout symptoms.

**Results:**

The presence of a high need for recovery and the presence of burnout symptoms were significantly associated with poor work-private life balance (OR 5.14 and 2.80, respectively), no quiet place to work (OR 3.23 and 2.00, respectively) and being worried about long-term work situation (OR’s increasing with increasing degree of worries). Being able to discuss the worries with the supervisor was only significant with a decreased risk of burnout symptoms for the lowest level of discussability with the supervisor. Following factors were not associated with both mental health outcomes: professional status, being worried about short-term work situation, taking care of ill or old people, number of children < 12yrs. at home and having a family member at risk for Covid-19.

**Conclusions:**

When working at home special attention should be paid to work-private life balance and the presence of a quiet place to work. Additionally, in the case of mandatory home work in university personnel, specific worries about long-term work situation should be tackled. Universities and/or governments should provide measures to ensure an extension of research deadlines and, if applicable, job security.

## Background

During the first wave of the Covid-19 pandemic in 2020, a total lockdown of universities was implemented by the government in Belgium. University staff was required to work at home. The mandatory shift from the office workplace to the home environment in a period where the home environment was prone to additional covid related stress factors could cause unforeseen implications on the mental well-being of the involved staff. From an occupational health point of view this caused several challenges. First of all, the physical accessibility of the occupational health service got lost: the occupational health physician was no longer available on site and the health examinations could no longer take place. Secondly, the staff had to master a completely new way of working (e.g. online teaching) in a short period of time, in an environment that originally was not designed for work (the home environment). The latter also being impacted by the ongoing pandemic: other family members at home due to the general lockdown, small children at home due to the closing of schools, possible presence of family members who were risk patients for Covid infection.

To meet the first challenge (ensuring occupational health surveillance in times where no presence on site was possible) an online questionnaire to assess mental health was developed to send to all university staff. This enabled to carry out our occupational health screening duties and to identify persons at risk for burn out and persons needing additional support or referral.

To meet the second challenge (the unusual working environment in that particular time period) specific home environment factors related to the mandatory homework (such as the presence or absence of a quiet environment, work-private life balance, additional caregiving tasks at home, …) were asked for as well. Indeed, the question could be raised if this particular work situation with its mandatory character induced specific risk factors for the mental well-being of the involved staff.

Therefore, the aim of this study was to identify factors associated with the mental health state of the university staff during their mandatory home work. This was an exploratory study using data collected within the framework of daily occupational health practice. No hypothesis testing was performed.

From a preventative point of view, monitoring mental well-being should enable to detect reduced well-being in a very early stage, in order to take preventive measures to prevent evolution to mental disorders. Need for recovery is considered to be a sensitive indicator of reduced well-being [1]. The concept of need for recovery is concordant with the cognitive activation theory of stress. In this theory stress response is defined as an alarm in a homeostatic system, producing neurophysiological activation. The activation can be reduced by coping mechanisms, triggered by the same alarm. If the coping mechanisms are inadequate to reduce the activation level, a certain aroused activation level remains. If sustained this may lead to adverse health effects [2]. In an occupational setting, fatigue experienced during or after a day’s work can lead to long term adverse health effects, when there is insufficient time to recover from this fatigue in between two work periods [3]. Increased need for recovery was shown to be a predictor for psychosomatic complaints and emotional exhaustion [4, 5]. As the need for recovery can be considered as an early indicator for the development of long-term adverse health effects, the need for recovery constitutes an important health outcome parameter when assessing mental well-being of employees.

A high need for recovery can be considered as an early indication of a (temporary) condition of exhaustion. If sustained and if no sufficient recuperation is possible, this can lead to severe exhaustion [6]. Burnout refers to a mental disorder that is characterized by severe mental exhaustion among those who cannot cope without the help or assistance of others [7]. Burnout could be considered as a possible next step subsequent to a sustained state of high need for recovery, and should be taken into account, when considering mental well-being of employees.

Earlier research suggested that need for recovery was associated with age [8, 9] and gender [8, 10]. Therefore, both factors were taken into account.

Family and social life factors outside work were also included in the study, as it was shown that family and social life stressors could influence psychosocial health leading to fatigue [8, 11, 12]. It could be hypothesized that within the context of working at home these factors could become more important within the framework of psychosocial well-being, because these factors are now becoming part of the (psychosocial) work environment.

It could be argued that the absence of a quiet workplace induces a situation where it is difficult to concentrate and could be an additional stressor. Having a quiet place to work at home or not was also taken into account.

Given the specific situation of university staff, comprising junior researchers with precarious employment statutes, who were concerned about not being able to finish their PhD thesis on time (and hence jeopardizing their funding for scholarship), these specific worries were included in the study.

Earlier studies showed a significant relationship between work-private life balance and mental health outcomes [13, 14] and was considered as well.

## Materials and methods

### Study design

The study aimed to identify factors associated with the mental health state of the university staff during their mandatory home work.

This was an exploratory study using cross-sectional questionnaire data collected within the framework of daily occupational health practice. The data were anonymized and analysed post hoc.

### Study population and data collection

All 1553 researchers and staff of a civic university in Flanders, Belgium, were addressed to fill out an online questionnaire. Employees were free to participate. At the occasion of the invitation all subjects were informed that the collected data could be used for post hoc scientific analyses. Eventually, 702 of them (45.2% response rate) returned the questionnaire.

Data collection took place from May 19th 2020 up to June 22nd 2020. This was approximately the third month of the first lockdown of the universities, which was implemented from March 14th 2020 and was still ongoing. At that time, the prospects for the restart of the normal activities at the universities were unclear.

### Outcome variables

Mental well-being was assessed by two health outcome parameters.

### Need for recovery

The subjective need for recovery is an indicator of fatigue at work and can be measured by ‘The need for recovery scale’. This scale was proven to be a reliable instrument [6, 15]. ‘The Need for Recovery Scale’ questionnaire comprises 11 dichotomous (yes/no) items [16]. To facilitate interpretation within the specific framework of homeworking, clarifications have been added in two items. Table 1 lists the 11 items of ‘The Need for Recovery Scale’ used in this study and their scores.

**Table 1:** The 11 constituent items of ‘The Need for Recovery scale’ [16]. Clarifications (in italic) have been added in items 7 and 9 to facilitate interpretation within the specific framework of homeworking

Item	answer (coding)
1. I find it hard to relax at the end of a working day	yes (1) / no (0)
2. At the end of a working day, I feel really worn-out	yes (1) / no (0)
3. My job causes me to feel rather exhausted at the end of a working day	yes (1) / no (0)
4. Generally speaking, I still feel fresh after supper	yes (0) / no (1)
5. Generally speaking, I am able to relax only on my second day off	yes (1) / no (0)
6. I have trouble concentrating in the hours off after my working day	yes (1) / no (0)
7. I find it hard to show interest in other people when I just came home from work *(in case of home work*: *“I find it hard to show interest in other people when I just stopped my teleworking at home”)*	yes (1) / no (0)
8. In general, it takes me over an hour to feel fully recovered after work	yes (1) / no (0)
9. When I get home, people should leave me alone for some time *(in case of home work*: *“When I have stopped my teleworking at home, people should leave me alone for some time”)*	yes (1) / no (0)
10. After a working day I am often too tired to start other activities	yes (1) / no (0)
11. During the last part of the working day I sometimes cannot perform optimally my job due to fatigue	yes (1) / no (0)

The need for recovery scale was computed by summing up the scores of the 11 constituent items, resulting in a score ranging from 0 to 11. Higher scores indicate a higher degree of need for recovery after work. Earlier research based on long term health effects enabled to determine a cut-off point for a high need for recovery: a need for recovery score higher than 5 indicates the presence of a high need for recovery [17].

Cronbach’s alpha of the scale was 0.83 (inter-item correlations 0.16–0.53), which was comparable to reliability reported earlier [18].

### Burnout symptoms

Burnout symptoms were assessed by using the 23 Core Symptoms questions on exhaustion, mental distance, cognitive impairment and emotional impairment of the Burnout Assessment Tool (BAT-C) [19]. Table 2 lists the 23 Core Symptoms of the Burnout Assessment Tool.

**Table 2:** The 23 Core Symptoms of the Burnout Assessment Tool [19]. For each item five answer possibilities were given: never (1), rarely (2), sometimes (3), often (4) and always (5)

1. At work, I feel mentally exhausted
2. Everything I do at work requires a great deal of effort
3. After a day at work, I find it hard to recover my energy
4. At work, I feel physically exhausted
5. When I get up in the morning, I lack the energy to start a new day at work
6. I want to be active at work, but somehow I am unable to manage
7. When I exert myself at work, I quickly get tired
8. At the end of my working day, I feel mentally exhausted and drained
9. I struggle to find any enthusiasm for my work
10. At work, I do not think much about what I am doing and I function on autopilot
11. I feel a strong aversion towards my job
12. I feel indifferent about my job
13. I’m cynical about what my work means to others
14. At work, I feel unable to control my emotions
15. I do not recognize myself in the way I react emotionally at work
16. During my work I become irritable when things don’t go my way
17. I get upset or sad at work without knowing why
18. At work I may overreact unintentionally
19. At work, I have trouble staying focused
20. At work I struggle to think clearly
21. I’m forgetful and distracted at work
22. When I’m working, I have trouble concentrating
23. I make mistakes in my work because I have my mind on other things

The Core Symptoms score was calculated according to the instructions resulting in a score ranging from 1 to 5. The cut-off value for employees was used to distinguish between no burnout symptoms (< 2.59) and presence of burnout symptoms (≥ 2.59) [7].

Cronbach’s alpha of the scale was 0.95 (inter-item correlations 0.20–0.78), which was comparable to reliability reported earlier [19].

### Considered factors

Gender and age were asked for in the questionnaire. The subjects were divided into two age groups: older workers (45 years or older) and younger workers (younger than 45 years), according to the definition of the World Health Organization [20].

Professional status was considered as well. The subjects were divided into five categories: academic staff tenured, academic staff untenured, PhD students, non-academic staff tenured, non-academic staff untenured.

Following family related factors were taken into consideration: taking care of ill or old people (yes / no), family member at risk for Covid-19 (yes / no), number of children < 12yrs. at home (0 / 1 / 2 / ≥3).

A specific home work factor was asked for by a single question: “Do you have a workplace where you can work quietly?” (yes / no).

The specific worries of the university staff were assessed by the following three question: “Are you worried about your short-term work situation?”; “Are you worried about your long-term work situation?”; “Can you easily discuss your worries about your current/future work situation with your supervisor?”. Each question had the following response categories: to a large extent; to a very large extent; somewhat; to a small extent; to a very small extent.

Work-private life balance was asked for by one question: “Do you have a good balance between your work and your private life?” (yes / no).

### Statistical analyses

All data analyses were performed using IBM SPSS Statistics, version 28.0.1.1.

All considered variables were described by number and percentage.

Differences in outcome prevalences between the considered groups were tested by the Chi-square test.

Binomial stepwise forward conditional multivariable logistic regression analyses were used to calculate the odds ratios and their 95% confidence intervals for the presence of a high need for recovery and the presence of burnout symptoms. For the stepping method criteria, the p value for including a variable was set at 0.05 and the p value for excluding a variable at 0.10. In each model age category (older vs. younger workers), gender (women vs. men), professional status (reference: tenured academic staff), work private life balance (poor vs. good), taking care of ill or old people (yes vs. no), having a family member at risk for Coronavirus at home (yes vs. no), quiet place to work (yes vs. no), worried about short-term work situation (reference: to very small extent), worried about long-term work situation (reference: to very small extent), worries easily discussable with supervisor (reference: to very large extent) were taken into account.

To prevent the occurrence of multicollinearity correlations between all independent variables were checked beforehand by examining the correlation matrix and no correlation was found higher than 0.80 [21]: the highest correlation (Spearman’s Rho = 0.68) was found between the two ‘worrying’ variables, all other correlations were ≤ 0.35.

## Results

Distributions of personal, family related and work related factors of the total study population are summarized in Table 3.

**Table 3:** Personal, family related and work related factors of the study population

Variable	n	*%*
age category		
< 45 yr.	505	*71.9*
≥ 45 yr.	197	*28.1*
gender		
men	248	*35.3*
women	454	*64.7*
professional status		
academic staff tenured	121	*16.5*
academic staff untenured	196	*26.7*
PhD students	126	*17.1*
non-academic staff tenured	108	*14.7*
non-academic staff untenured	184	*25.0*
taking care of ill or old people		
yes	98	*15.9*
no	518	*84.1*
family member at risk for Coronavirus		
yes	155	*25.2*
no	461	*74.8*
number of children < 12yrs. at home		
0	399	*64.8*
1	85	*13.8*
2	103	*16.7*
≥ 3	29	*4.7*
quiet place to work		
yes	518	*84.1*
no	98	*15.9*
worried about short-term work situation		
to a very small extent	221	*35.9*
to a small extent	175	*28.4*
somewhat	140	*22.7*
to a large extent	51	*8.3*
to a very large extent	29	*4.7*
worried about long-term work situation		
to a very small extent	179	*29.1*
to a small extent	164	*26.6*
somewhat	151	*24.5*
to a large extent	80	*13.0*
to a very large extent	42	*6.8*
worries easily discussable with supervisor		
to a very small extent	59	*9.6*
to a small extent	80	*13.0*
somewhat	172	*27.9*
to a large extent	214	*34.7*
to a very large extent	91	*14.8*
work-private life balance		
good	424	*68.8*
poor	192	*31.2*

Table 4 shows the results of the considered outcome variables for the total study population.

**Table 4:** Outcome variables for the total study population

Variable	n	*%*
need for recovery		
low need for recovery	407	*61.6*
high need for recovery	254	*38.4*
burnout symptoms		
no burnout symptoms	506	*79.7*
burnout symptoms	129	*20.3*

Prevalences of high need for recovery and burnout symptoms by considered factor are given in Table 5. Significant differences were found in work-private balance and all considered work related factors. The prevalences found showed a logical increasing trend. Noteworthy was the finding that the highest prevalence of high need for recovery was found in the youngest group, albeit not on statistically significant level (p = 0.101). Prevalence of high need for recovery was significantly higher in women; prevalence of burnout symptoms were also higher in women, but the difference did not reach statistical significance (p = 0.072).

**Table 5:** Presence of high need for recovery (NFR) and burnout symptoms (BURNOUT) by considered factor

Variable	NFR	BURNOUT
	n (%)	n (%)
age category		
< 45 yr.	161 (40.4)	97 (21.6)
≥ 45 yr.	63 (33.5)	32 (17.3)
gender		
men	69 (30.3)**	36 (16.4)
women	185 (42.7)**	93 (22.4)
professional status		
academic staff tenured	45 (42.1)	20 (19.4)
academic staff untenured	68 (38.9)	33 (19.5)
PhD students	43 (39.4)	22 (21.2)
non-academic staff tenured	40 (40.0)	17 (17.7)
non-academic staff untenured	58 (34.1)	37 (22.7)
work-private life balance		
good	102 (24.1)***	54 (12.7)***
poor	126 (65.6)***	70 (36.5)***
taking care of ill or old people		
yes	31 (31.6)	21 (21.4)
no	197 (38.0)	103 (19.9)
family member at risk for Coronavirus		
yes	54 (34.8)	34 (21.9)
no	174 (37.7)	90 (19.5)
number of children < 12yrs. at home		
0	139 (34.8)	84 (21.1)
1	35 (41.2)	14 (16.5)
2	42 (40.8)	18 (17.5)
≥ 3	12 (41.4)	8 (27.6)
quiet place to work		
yes	165 (31.9)***	89 (17.2)***
no	63 (64.3)***	35 (35.7)***
worried about short-term work situation		
to a very small extent	49 (22.2)***	18 (8.1)***
to a small extent	65 (37.1)***	29 (16.6)***
somewhat	66 (47.1)***	40 (28.6)***
to a large extent	27(52.9)***	21 (41.2)***
to a very large extent	21 (72.4)***	16 (55.2)***
worried about long-term work situation		
to a very small extent	33 (18.4)***	14 (7.8)***
to a small extent	54 (32.9)***	21 (12.8)***
somewhat	70 (46.4)***	35 (23.2)***
to a large extent	44 (55.0)***	32 (40.0)***
to a very large extent	27 (64.3)***	22 (52.4)***
worries easily discussable with supervisor		
to a very small extent	31 (52.5)***	22 (37.3)***
to a small extent	40 (50.0)***	21 (26.3)***
somewhat	73 (42.4)***	45 (26.2)***
to a large extent	64 (29.9)***	30 (14.0)***
to a very large extent	20 (22.0)***	6 (6.6)***

* p < 0.05 ** p < 0.01 *** p < 0.001

The final multivariable logistic regression models for the presence of a high need for recovery and burnout symptoms are shown in Table 6.

**Table 6:** Final multivariable logistic regression models (n = 616) for the presence of a high need for recovery (NFR) and burnout symptoms (BURNOUT).

	NFR	BURNOUT
Variable	OR (95% CI)	OR (95% CI)
poor work-private life balance	5.14 (3.46–7.61)***	2.80 (1.79–4.36)***
no quiet place to work	3.23 (1.95–5.34)***	2.00 (1.18–3.39)*
worried about long-term work situation		
to a very small extent	1	1
to a small extent	2.18 (1.27–3.73)**	1.49 (0.71–3.10)
somewhat	3.31 (1.93–5.67)***	2.57 (1.27–5.17)**
to a large extent	4.08 (2.16–7.70)***	4.95 (2.34–10.51)***
to a very large extent	4.98 (2.19–11.34)***	6.30 (2.61–15.24)***
worries discussable with supervisor		
to a very large extent		1
to a large extent		1.87 (0.70–4.96)
somewhat		2.63 (1.00-6.92)
to a small extent		2.25 (0.78–6.47)
to a very small extent		4.56 (1.56–13.30)**

* p < 0.05 ** p < 0.01 *** p < 0.001

Three factors were strongly significant associated with the presence of a high need for recovery: poor work-private life balance (OR 5.14), no quiet place to work (OR 3.23) and being worried about long-term work situation (OR’s increasing with increasing degree of worries).

Four factors were strongly significant associated with the presence of a burnout symptoms: poor work-private life balance (OR 2.80), no quiet place to work (OR 2.00), being worried about long-term work situation (OR’s increasing with increasing degree of worries), and being able to discuss the worries with the supervisor (only significant for the lowest level of discussability with the supervisor).

Following factors were not associated with both mental health outcomes: professional status, being worried about short-term work situation, taking care of ill or old people, number of children < 12yrs. at home and having a family member at risk for Covid-19.

## Discussion

The prevalences of high need for recovery and burnout symptoms in the current study population were 38.4% and 20.3% respectively.

The prevalence of high need for recovery found in the current study was similar to the percentage found in an earlier study in workers in the Flemish public sector (37.7%) [8]. However, in the current study younger workers tended to have a higher need for recovery than older worker, which is in contrast with earlier reports [8, 9]. This inconsistency could be explained by the fact that in the current population the older employees are in a more “comfortable” occupational and family related position than the younger ones. It could be argued that the older employees are more likely to occupy a permanent position as a member of the higher academic staff, while the younger are more likely to have a more precarious employment status (such as temporary PhD scholarship) and to have a less “consolidated” family life situation.

Compared to a random sample from the Flemish workforce representative of age, gender and industry [7] our results showed a lower prevalence of burnout symptoms (20.3% vs. 25.6%). The difference could be due to a different constitution of the study population. The current population was a specific university population, comprising a substantially higher proportion of female employees (64.7% vs. 45.7%) with a lower mean age (38.8 yrs. vs. 41.3 yrs.) and, because this concerns a population of university workers, one could expect a higher proportion of higher educated employees.

Having a poor work-private life balance was by far the most important factor for the two considered mental health outcome parameters, both in univariable and multivariable analyses. In the multivariable models the odds ratios reached 5.14 for having a high need for recovery and 2.80 for having burnout symptoms. These findings are in accordance with the findings of earlier studies. In a representative sample of the working population, employees with high work life conflicts showed a high risk of anxiety and depression, lack of energy and optimism, headaches, sleep disorders and fatigue [13]. Similarly, in a sample of academics, poorer work-life balance was associated with perceived job stress [14]. Our findings are also in line with the theoretical framework of need for recovery: if a poor work-life balance does not enable to recover sufficiently after work, the need for recovery will increase, eventually leading to a high need for recovery.

Having no quiet place to work was significantly associated with the presence of burnout symptoms and highly significant associated with a high need for recovery, with odds ratios of 2.00 and 3.23 respectively. To our knowledge these associations have not been reported before. Earlier studies have shown that an “open plan” working place, also a situation where employees have less privacy to concentrate on their work, can induce increased stress and other adverse health effects [22]. A possible explanation could be that the absence of a quiet workplace induces a situation where it is difficult to concentrate, which is an additional stressor, consuming extra energy and resulting in a higher need for recovery and subsequently burnout.

Being worried about short-term work situation and being worried about long-term work situation were both significantly associated with need for recovery and burnout in univariable analyses (p < 0.001). However, in the multivariable analyses only being worried about long-term work situation was significantly associated with the presence of a high need for recovery (odds ratios ranging from 2.18 to 4.98) and burnout symptoms (odds ratios ranging from 1.49 to 6.30). For both outcome parameters the OR’s showed a logical gradient: the more worried about long-term work situation, the higher the risk for the presence of a high need for recovery and burnout symptoms.

No literature was found on the relationships of being worried about long-term work situation with need for recovery and burnout. However, it could be hypothesized that job insecurity can be considered as a worry of the employee about his current employment. Therefore, we considered job insecurity as an alternative for being worried about long-term work situation in our search for literature. Job insecurity has been found to be related to high need for recovery in earlier studies. In a study in employees from various sectors, job insecurity was found to be positively related to need for recovery [23]. This was confirmed in subsequent research, where in a population of public sector employees job insecurity was significantly associated with a high need for recovery [18]. Job insecurity was also shown to be significantly associated with exhaustion, a core component of burnout [23, 24] and positively associated with burnout [25, 26]. In the specific case of university personnel the worries relate to not meeting the research deadlines due to the closure of research labs, and hence jeopardizing their scholarship, and subsequently resulting in a postponing or even a cancelling of their future academic career. In that regard our results seem to corroborate earlier findings. A possible explanation could be that being worried about long-term work situation is a stressor that consumes extra energy, resulting in a higher need for recovery, and subsequently to burnout.

No significant association between worries being discussable with the supervisor and need for recovery was found. This in concordance with earlier research where social support from supervisor was also not associated with need for recovery [18].

Our results showed only a significant association between worries being discussable with the supervisor and burnout if the worries were discussable to a very small extent. Earlier research showed that fewer sources of support go along with a significantly increased risk of burnout symptoms [27] and that supervisor support was negatively associated with burnout [28]. It was also suggested that supervisors can help university employees lower emotional exhaustion by reducing the degree of the perceived uncertainties [29]. Our results indicated a less pronounced negative relationship (only if the worries were discussable to a very small extent). This could be due to the very specific situation of the current study population. It could be argued that in mandatory home work implemented without any preparation, contacts with the supervisors are not institutionalized properly from the beginning and are less a factor of concern.

No significant associations were found with professional status. Apparently, the mechanisms for having a high need for recovery or burnout symptoms are not related to professional status, but rather to factors that interfere with energy levels.

Factors related to the family situation (taking care of ill or old people, number of children < 12yrs. at home and having a family member at risk for Covid-19) were not associated with both mental health outcomes, neither in univariable and multivariable analyses. This is in accordance with earlier research, where no significant associations were found between need for recovery and taking care of ill or old people, number of children < 12yrs. at home [8, 18]. This could be an indication that people are more likely to cope with the (normal) familial situation, than with external stressors. Possibly, taking care of family members is more close to our ‘natural’ behaviour (our genetic program is designed to maintain the species, which involves the ability to taking care of family members) than dealing with the artificial institution of work (for which our genetic program has not been designed).

The strongest associations were found for the presence of a high need for recovery. This could be explained by the fact that a high need for recovery is a sensitive indicator of reduced well-being in its very early stage [1], while the presence of burnout symptoms reflects already the presence of a long term health effect, making this measure less sensitive than the need for recovery. It could be expected that the higher sensitivity of the need for recovery will make it more ‘vulnerable’ for certain risk factors, resulting in higher effect measures (higher odds ratios).

This study is subject to some limitations. First of all, this study has a cross-sectional design, not allowing to draw reliable conclusions on causal relationships between the considered factors and health outcomes. The direction of the causal relationship cannot be determined with the current study design; a longitudinal study is needed to allow causal interpretations. Furthermore, it cannot be excluded that people with a poor mental health (high need for recovery or burnout symptoms) might perceive certain factors as worse than people with a good mental health. The associations found could be inflated by the state of mind of the participants.

Secondly, although a few home environment factors have been asked for, it is clear that many factors in the (psychosocial) environment of the home setting (e.g. interpersonal relationship issues, …) could be of influence on the mental health of the home working employee. Mandatory home work could force the employee to work in a “toxic” familial setting and hence influence his/her mental health and productivity.

Thirdly, job related factors (quantitative demands, emotional demands, …) were not asked for and could possibly have an influence as well [18]. It is not sure if they would overrule the significant associations found in this study or only be additional significant factors.

Fourthly, the rather low response rate (45.2%) could be subject to selection bias. This is relevant when there is a difference between responders and non-responders in mental health outcomes and influencing factors and when the reason for nonresponse is correlated with the variables investigated. However, low response rates need not necessarily lead to biased results. Bias is more likely to be present when examining a simple univariable distribution than when examining the relationship between variables in a multivariable model [30].

The study design of the current study did not allow to monitor changes. Future (longitudinal) research should focus on changes in mental well-being when a mandatory shift from workplace office to home office is implemented. Simultaneously, the influence of the changing environmental factors when moving from the workplace environment to the home environment should be explored. Impact of “non-traditional” occupational factors linked to the home environment should be studied as well, with special focus on the psychosocial home environment.

## Conclusions

In mandatory home work, poor work-private life balance, having no quiet place to work at home and being worried about long-term work situation were important factors for having a high need for recovery and burnout symptoms. Being able to discuss worries with the supervisor tended to reduce burnout symptoms.

Mental disorders are of growing concern in the working population. In Belgium, mental disorders constitute the main cause of long-term sickness absence (> 12 months) (37.2% in 2021) and its part is increasing [31]. Prevention on the work floor is of the utmost importance. Monitoring mental well-being and taking preventive measures in an early phase are therefore necessary.

In daily occupational health practice, monitoring the need for recovery enables to identify persons at risk in a very early stage of mental unwell-being. Additionally, two questions can offer the practitioner a good starting point for further counselling, if necessary: ‘do you have a good work-private life balance?’ and ‘do you have a quiet place to work?’.

When working at home special attention should be paid to work-private life balance and the presence of a quiet place to work. Additionally, in the case of mandatory home work in university personnel, specific worries about long-term work situation should be tackled. Universities and/or governments should provide measures to ensure an extension of research deadlines and, if applicable, job security.

Although this study was carried out in a specific population, it could be argued that the main findings could also be relevant for other occupational groups that perform similar work at home (intellectual workers, administrative workers, …). Poor work-private life balance, having no quiet place to work and job insecurity are factors that are not exclusively linked to university staff and by extension also not to home work.

## Data Availability

The data presented in this study are available on reasonable request from the corresponding author.
